# Elements of Physics, or Natural Philosophy, General and Medical, Explained Independently of Technical Mathematics; and Containing New Disquisitions and Practical Suggestions

**Published:** 1831

**Authors:** 


ART. VI.—*Elements of Physics, or Natural Philosophy, General and Medical, explained independently of Technical Mathematics; and containing new disquisitions and practical suggestions*—By NEIL ARNOTT, M. D., of the Royal College of Physicians.—First American, from the third London edition, with additions. By ISAAC HAYS, A. M., M. D., &c.—Philadelphia: Carey, Lea & Carey.—pp. 582.—(*Concluded from last number.*)

Our author's remarks on some of the common instruments in use, deserve attention. Many practitioners make use of these ordinary contrivances without having an adequate idea of the mechanical principles of their action. We heartily unite with him in the recommendation of the self-adjusting truss, as originally invented in England by Salmon and Ody, and as altered and patented in the United States by Dr. Hull. The shops in this city present many varieties of this instrument; but in all of them, the great principle of pressure on the hernial aperture, and on the back, are maintained, and we do not think there is much choice between those with a convex, a flat or a concave pad.

We commend to the attention of our readers, the remarks on tooth-drawing.

*The Obstetric forceps.*—As the blades beyond the joint or fulcrum are longer than the handles, the pressure on the head included in them is less than that exerted by the hand that uses them; but its degree should always be kept present to the mind of the operator.

*The vectis*, or lever, used instead of the forceps just mentioned, is a very dangerous instrument in unskilful hands. In fact, whenever it is used as a lever, in the common acceptation of the term, it is a piece of unskilful cruelty. If we suppose any part of the pelvis to be made the fulcrum of this lever, the soft parts between the bone and the instrument are bruised, not only with the whole force of the hand, but with twice or three times as much, if the resistance be so much nearer to the fulcrum than to the hand. The instrument is only safely used when the operator makes one of his hands the fulcrum, and uses the other as the power, or maks one hand answer both purposes; and then there is a resemblance between the action of the vectis and of a hook.

*The Levator*, or lever, for raising the broken and depressed portion of the skull in trepanning, has a jointed fulcrum attached to it. Care should be taken to place this fulcrum where its pressure cannot be injurious.

*The circular* or *crown saw* of the trephine, should be worked with a quick motion and gentle pressure, for the reason given at page 178, when treating of cutting instruments. The object is thereby sooner and better attained, and the head of the patient is less shaken.

For the same reason, there is less jarring, and an easier division of the bone in amputations, when a light and quick motion of the straight saw is used.

In using the amputation knife, the speed, neatness, and success of the operation, are all favored by blending the drawing or saw motion of the knife, with the pressure towards the bone.

These last observations are of a hundred similar, which might be made to prove the vital importance to a surgeon, of having familiarity with the use of tools or instruments. Perhaps a person cannot better acquire this than by practising, while young, some amusing work of carpentry. Manual dexterity, and a little readiness at mechanical contrivance, so frequently prove of importance to persons in all stations, that a great defect in systems of general education, is the not cultivating them with greater attention. If a handless or awkward man embrace the medical profession, and unfortunately attempt to practice surgery or midwifery, although possessed of brilliant intellect, he will very often fail where another would succeed.

*The Tooth Key* is an instrument found in the hands of most persons who pretend even to the lowest degree of skill in the healing art: and there is perhaps scarcely a day passing, in which teeth are not broken, and jaws splintered, and gums bruised even to sloughing, by the unskilful or awkward use of it. The common tooth key may be regarded in the light of a wheel and axle; the hand of the operator acting on two spokes of the wheel to move it, while the tooth is fixed to the axle by the claw, and is drawn out as the axle turns. The gum and alveolar process of the jaw, form the support on which the axle rolls. The common errors in tooth-drawing by the key, are these:

1st. Turning the key towards that side, where the adjoining teeth are so close that the tooth to be drawn cannot pass without either breaking one of them or being broken itself. Sometimes two teeth are moved instead of one.

2d. Neglecting the natural inclination of the tooth. By winding the tooth round in the direction in which it already inclines, and in accordance with a bend which is generally found in it, the operation is easy and safe; but if it be drawn in the opposite way, it not unfrequently breaks or splinters the part of the jaw-bone in which it sticks.

3d. If the tooth-claw be blunt, its point may slip upon the tooth, so as to produce an action which is very apt to break the tooth.

4th. Unless the axle or fulcrum of the key be made to rest as evenly as possible on the gum, it will tear, or very much injure the gum. It should rest, if possible, over the part of the bone in which the tooth is set, for otherwise—as when a back tooth is drawn with the instrument resting on a part considerably anterior to it—the twist produced is painful, and there is danger of splintering.

A man whose studies or reflection have suggested these remarks, and who then operates leisurely a few times on the dead subjects, will often be able to give instant and safe relief to most intense suffering. And it is hardly excusable in any medical man, who may be placed where a dentist cannot be procured, to neglect acquiring a talent so easy.

Some dentists pull teeth directly out, by a strong forceps, made for the purpose; others use a forceps in the manner of a tooth key, by resting one side of it on the gum as a fulcrum. In this case the resting side is formed like the bolster of a tooth key. But much more in all cases depends on the dexterity of the operator, than on the form of the instrument.

*Steel Trusses for ruptures* are one of the blessings to suffering humanity, which modern ingenuity has supplied. From the unhealthy employment of some men in society, and the early dissipation or unnatural modes of life of others, debilitated constitutions are frequent, and are often transmitted to offspring; and one of the lamentable effects is, that weakness of the flesh forming the sides of cavities, which at particular points allows the protrusion of the living parts from within, so as to form tumors under the skin. The occurrence is called *hernia*, or *rupture:* the most common hernia is that of the intestines, through the groins.

Formerly this occurrence disabled for life. A man who had hernia was discharged from the army or navy; he could not ride on horseback, or take usual exercise; he could not lift a weight, and in a word, he often became a miserable burden to himself and others.—Now, by fitting the pad of a good steel truss to the part, the rupture is as perfectly restrained, as if the hand of a skilful surgeon were constantly there. The truss may be put on and off, with as little reflection or trouble as a part of the ordinary dress, and the man becomes again almost as fit for all the duties of life as if he were without his ailment.

The old form of the steel truss was that of a half or three quarter hoop, so bent and tempered, that when put upon the patient, one end, which had a pad upon it, pressed with a given force on the opening by which the rupture protruded. The defects in this kind of truss are, the difficulty of making it to fit exactly; its being rather troublesome to put on and off; and its pressing disagreeably all round the body.

The other kind of truss, free from these defects, consists of a little more than half a hoop, with a pad at each end: one of the pads supports the weakness, and the other rests upon the centre of the back, to bear all the strain there, while the hoop itself reposes loosely on the side of the body. This truss may be called self-adjusting, for it almost falls into its place of itself, and needs no fastenings; the same truss fits all persons of one size, whatever their shape; and the strength may be adjusted by changing the number of plates in the spring-hoop.

*Tourniquets, crutches, splints,* &c. &c., are so simple in all respects as not to merit special notice here.

The third part is devoted to the doctrine of fluids. We cannot refrain from extracting the following paragraphs.

As in the animal body, from even the minutest point, a little vein endowed with living power, takes the blood which has just brought life and nutriment to the part, and delivers it into a larger vein, whence it passes into larger still, until at last, in the great reservoir of the heart, it meets the blood returned from every part of the body: so in this terrestrial globe, where the magic-moving power is simply fluid seeking its level, from every point of the surface, does the rain, which falls to sustain vegetable and animal life, and to renovate nature, glide into a lower bed, and from thence into a lower still, until the countless streams, after every variety of course, combine to form the swelling rivers, which return the accumulated waters into the common reservoir of the ocean. In the living body, the arteries carry back the blood with renewed vitality, to every point whence the veins had withdrawn it, and so complete the circulation; and in what may be called the living universe, the circulation is completed by the action of heat, and of the atmosphere, which, from the extended face of the ocean, raise a constant exhalation of watery vapour of invisible purity, which the winds carry and deposit as rain or dew on every spot.

A very slight declivity suffices to give the running motion to water. Three inches per mile, in a smooth, straight channel, gives a velocity of about three miles per hour. The Ganges, which gathers the waters of the Himalaya mountains, the loftiest in the world, at eighteen hundred miles from its mouth, is only eight hundred feet above the level of the sea—that is, about twice the height of St. Paul's Church in London; and to fall these eight hundred feet, in its long course, the water requires more than a month. The great river Magdalena, in South America, running for a thousand miles between two ridges of the Andes, falls only five hundred feet in all that distance. Above the commencement of the thousand miles, it is seen descending in rapids and cataracts from the mountains. The gigantic Rio de la Plata has so gentle a descent to the ocean, that in Paraguay, at the distance of fifteen hundred miles from its mouth, large ships are seen, which have sailed against the current all the way, by the force of the wind alone: that is to say, which on the beautifully inclined plane of the stream, have been lifted even by the soft wind, to an elevation greater than of our loftiest spires.

A small lake or extensive mill-pond, with very uneven bottom, if suddenly emptied by a sluice or opening at its lowest part, would exhibit a vast number of pits or pools of various size and shape left among its inequalities. But supposing rain to continue falling, or frequently to occur, a remarkable change would soon be effected. In consequence of each pool discharging over its lower part, that is, sending out a streamlet, either into another lower pool, or into a channel leading directly to the sluice or opening, there would be a constant wearing of the part or side over which the water was running, that is to say, a deepening of the breach or channel, and the water in the pool would be consequently becoming shallower, while at the same time the bottom would be filling up with the sand or mud washed down by the rain, from the elevations around; and these two operations continuing, the pool would at last altogether disappear. By this change going on in every pool through the whole of the emptied mill-pond, the bottom would at last exhibit only a varied or undulated surface of dry land, with a beautiful arrangement of ramifying channels, all sloping with a precision unattainable by art, to the general mouth or estuary.—The reason that in the supposed case, and in every other, a water course soon becomes so singularly uniform, both as to dimension and descent, is, that any pits or hollows in it are filled up by the sand and mud carried along in the stream, and deposited where the current is slack; while any elevations are worn away by the action of the more rapid current which accompanies shallowness.

The peculiarly interesting subject of fluid support is treated by our author with great ingenuity, and his observations on the support which the human body receives in water, ought to be understood by every one.

The art of swimming, which is of such universal importance, depends on this support; and even the best swimmer cannot but receive pleasure and instruction in the perusal of the paragraphs which we are about to extract. The life of many an individual might have been saved by a knowledge of this art so easily acquired, and so pertinaciously retained. We think that a father who neglects having his son made an expert swimmer, commits an error of no common magnitude in his education. So fully were the ancients persuaded of the importance of this acquisition, that no individual was permitted to enroll his name under the victorious eagles of a Roman army, who could not swim.—Our illustrious countryman, Dr. Franklin, has urged on us the necessity of educating our children in this pleasant, healthful and important art.

The human body, in an ordinary healthy state, with the chest full of air, is lighter than water.

If this truth were generally and familiarly understood, it would lead to the saving of more lives, in cases of shipwreck, and in other accidents, than all the mechanical life-preservers which man's ingenuity will ever contrive.

The human body with the chest full of air, is so much lighter than water, that it naturally floats with a bulk of about half the head above the water—having no more tendency to sink than a log of fir. That the person may live and breathe, then, it is only necessary to exert volition, so as to render the face the part which remains uppermost.

The reasons that so many people are drowned in ordinary cases, who might easily be saved, are the following:

1st. Their believing that continued exertion is necessary to keep the body from sinking, and hence their generally assuming the position of a swimmer, in which the face is downwards, and the whole head must be kept out of the water to allow of breathing. Now as a man cannot retain this position without continued exertion, he is soon exhausted, even if a swimmer, and if not, the unskilful attempt will scarcely secure for him even a few respirations. The body raised for a moment by exertion, above the natural level, sinks as far below when the exertion ceases; and the plunge, by appearing the commencement of a permanent sinking, terrifies the unpractised individual, and renders him an easier victim to his fate.

2d. From a fear that water entering by the ears may drown, as if it entered by the nose or mouth, a wasteful exertion is made to prevent it; the truth being, however, that it can only fill the outer ear, or far as the membrane of the drum, and is therefore of no consequence. Every diver or swimmer has his ears filled with water, and with impunity.

3d. Persons unaccustomed to the water, and in danger of being drowned, generally attempt in their struggle to keep their hands above the surface, from feeling as if their hands were tied while held below; but this act is most hurtful, because any part of the body kept out of the water, in addition to the face which must be so, requires an effort to support it, which the individual is supposed at the time incompetent to afford.

4th. Not having reflected, that when a log of wood or a human body is floating upright, with only a small portion above the surface, as at sea, every wave in passing must cover the head for a little time, but will again leave it projecting in the interval. The practised swimmer chooses this interval for breathing.

5th. Not knowing the importance of keeping the chest as full of air as possible; the doing which has nearly the same effect as tying a bladder of air to the neck, and without other effort will cause nearly the whole head to remain above the water. If the chest be once emptied, and if from the face being under water, the person cannot inhale again, the body is then specifically heavier than water, and will sink.

A very interesting case is given of death from compression of the chest, produced by sinking to a great depth, by which the body becomes really heavier than water, and will not again rise to the surface but for the exertion of swimming. It occurred in a stout negro, an excellent swimmer, who fell from a yard arm into the calm sea, a distance of eighty feet. The great velocity drove him deep into the sea at once. Stunned, most probably, by the fall, he was seen only to make a slight and ineffectual effort with his arms, "and was then seen gradually sinking for a long time afterwards, until he disappeared, as a black and distant speck, towards the unknown regions of the abyss." It is more than probable, that the renowned Sam Patch met his fate in a similar way, for it appears, at his last leap, he was more than usually intoxicated, and did not reach the water in his accustomed manner. The concussion was then so great, that he neglected the proper means for raising his body again to the surface.

We must be allowed to offer another extract.

It is not to be expected that every person should learn to swim; but every one who makes voyages, should have practised the easy lesson of resting in the water with the face out. The head, from the large quantity of bone in it, is a heavy part of the body, yet a little action of adjustment with the hands, easily keeps it uppermost; and there is an accompanying motion of the feet, called *treading the water,* not difficult to learn, which sustains the entire head above the surface. Perhaps the whole of the seventy passengers who were swallowed up on the sudden sinking of the Comet steam-boat near Greenock, in November, 1825, might have been saved in the boats which so soon went to their assistance, had they known the truth which we are now explaining.

In having to swim far, a man may rest on his back for a time, and resume his labour when he is somewhat refreshed.

So little is required to keep a man's whole head out of water, that many individuals, altogether unacquainted with what regards swimming or floating, have been saved after shipwreck, by catching hold of a few floating chips, or broken pieces of wood. An oar will suffice as a support to half a dozen people, if no one of the number attempts to keep more than his head out of the water; but from each wishing to have a good share of the security, it is often rendered less useful than it might be.

A common life preserver consists of strings of corks put round the chest or neck; or of an air-tight bag applied round the upper part of the body, and which can be filled by the person blowing into it through a valved pipe.

On the great rivers of China, where thousands of people find it more convenient to live in covered boats upon the water, than in houses on the shore, the younger male children have a hollow ball of some light material attached constantly to their necks, so that in their frequent falls overboard, they are not in danger.

Life boats have a large quantity of cork mixed in their structure; or of air-tight vessels of thin copper or tin plate, so that even when the boats are filled with water, a considerable part still floats above the general surface.

Swimming is much easier to quadrupeds than to man, because the common motion of their legs in walking and running is that which best supports them in swimming. Man is the most helpless creature in water. A horse can carry his rider with half the body out of the water; dogs that have never been in water, swim well on the first trial. Swans, geese, and water-fowls in general, are so bulky and light, by the great thickness of feathers under them, that they float upon the water like stately ships, moved by their webbed feet as oars.

A man in deep water may walk upon broken glass with impunity, because his weight is supported by the water.

But many men have been drowned in attempting to wade across the fords of rivers, from forgetting that the body is supported by the water, and does not press on the bottom sufficiently to give a sure footing against a very trifling current. A man therefore, carrying a weight on his head or shoulders, may safely pass a river, where without a load, he would be carried down in the stream.

Under the head of pneumatics, Dr. Arnott makes the following observations, after having alluded to the chemical elements of the atmosphere:

The atmospheric ocean is the great laboratory in which most of the actions of life go on, and on the composition of which they depend. A human being requires for breathing, a gallon of fresh air every minute, dying equally if deprived of it, or if confined to the same. All other animals also require fresh air, but in various proportions. And in the vegetable creation, the beautiful green leaf and delicate flower are merely broad and tender expansion of surface for the contact of the vivifying air. Animals give out to the atmosphere a substance which vegetables absorb; and vegetables, by the absorption, fit the air again for the use of animals; so that, upon the whole, in the various changes of nature, there is a perfect balancing of actions preserving the amospheric mass in a uniform state, constantly fit for its admirable purpose.

We know that this doctrine of compensation has of late years been stoutly denied. It has been asked, how is this supposed balance kept up during the winter, when of course the vegetable absorption of nitrogen is very much diminished, while no material change is effected in the animal consumption of oxygen? Late experiments have proved that the insect tribes so numerous in the summer time, are consumers of oxygen, and that the atmospheric air after having served their purposes of respiration, and returned through their spiracula, is deteriorated much in the same manner as it is after having passed thro' the lungs of the higher order of animals. This important observation removes, in a great measure, the obstacles to the belief of this beautiful supposition.

The author has exhibited in an interesting manner, the nature of atmospheric pressure on the human body.

*The animal body* is made up of solids and fluids, and is affected by the atmospheric pressure accordingly. There is a difficulty at first in believing that a man's body should be bearing a pressure of fifteen pounds on every square inch of its surface, while he remains altogether insensible to it; but such is the fact, and the reason of his not feeling the fluid pressure is its being perfectly uniform all around.—If a pressure of the same kind be even many times greater, such for instance, as fishes bear in deep water, or as a man supports in the diving bell, it equally passes unnoticed. Fishes are at their ease in a depth of water where the pressure around will instantly break or burst inwards almost the strongest empty vessel that can be sent down; and men walk on earth without discovering a heavy atmosphere about them, which, however, will instantly crush together the sides of a square glass bottle emptied by the air-pump, or even of a thick iron boiler, left for a moment by any accident, without the counteracting internal support of steam or air.

The fluid pressure on animal bodies, thus unperceived under ordinary circumstances, may be rendered instantly sensible by a little artificial arrangement. In water, for instance, an open tube partially immersed becomes full to the level of the water around it, and the water contained in it is supported, as already explained, by that which is immediately below its mouth: now a flat fish resting closely against the mouth of the tube, would evidently be bearing on its back the whole of this weight—perhaps one hundred pounds; but the fish would not thereby be pushed away, nor would it even feel its burden, because the upward pressure of the water immediately under it would just counterbalance the weight, while the lateral pressure around would prevent any crushing effect of the upward and downward forces. But if while the fish continued in the situation supposed, the hundred pounds of water were lifted from off its back by a piston in the tube, the opposite upward pressure of one hundred pounds would at once crush its body into the tube. At a less depth, or with a smaller tube, the effect might not be fatal, but there would be a bulging or swelling of the substance of the fish into the mouth of the tube. In air and on the human body, a perfectly analogous case is exhibited. A man without pain or peculiar sensation, applies his hand closely to the opening of a tube, or to the mouth of any vessel containing air, but the instant that the air is withdrawn from within the tube or vessel, the then unresisted pressure of the air on the outside, fixes the hand upon the vessel's mouth, causes the flesh to swell or bulge into it, and makes the blood ooze from any crack or puncture in the skin.

These last few lines describe the surgical operation of cupping: the essential circumstances of which are the application of a cup of glass with a smooth blunt lip, to the skin of any part, and the extraction, by a syringe or other means, of a portion of the air from within the cup. To some minds, the exact comprehension of this phenomenon may be facilitated, by considering the similar case of a small bladder or bag of India-rubber full of any fluid, and pressed between the hands on every part of its surface except one:—at that one part it will swell, and even burst if the pressure be strong enough. So in cupping, the whole body, except the surface under the cup, is squeezed with a force of fifteen pounds to the square inch, while in that one situation, the pressure is diminished according to the degree of exhaustion in the cup, and the blood consequently accumulates there. The application of a cup with exhaustion only, constitutes the operation called *dry-cupping*. To obtain blood, the cup is removed, and the tumid part is cut into by the simultaneous stroke of a number of lancet points; and the cup is afterwards used as before, so that the blood may rush forth under the diminished pressure. The partial vacuum in the cup may be produced either by the action of a syringe, or by burning a little spirit in the cup and applying it while the momentary dilation affected by the heat has driven out the greater part of the air. The human mouth applied upon any part becomes a small cupping machine, and formerly, in cases of poisoned wounds, was used as such. Our present perfect cupping glasses, of stronger and more permanent operation, are not yet always used, as they might be, to assist in removing the poison after the bites of rabid or venomous animals.

He tells us too, that atmospheric pressure tends to keep all the parts about the joints firmly bound together. Thus, when the shoulder or other joints are luxated, no empty space is left, as might be supposed, but the soft parts around are pressed in to fill up the space. In dislocation of the hip, the deep socket of the acetabulum acts like a cupping glass, and is filled partly with fluids, and partly with soft solids. In all the joints the bones are, by the means of atmospheric pressure, kept in such steady contact that they work without noise or jarring.

In speaking of the barometer, our author has fallen into an error, that considering his depth of learning, and extent of research, we should hardly have expected. He ascribes the honor of having made that ascent in a balloon, which raised him to the greatest elevation "to which man has ever ascended above the surface of his earthly habitation," to De Luc. Now every one knows that it was not De Luc, but Gay Lussac, to whom this merit is due. We can scarcely suppose this to be a slip of the pen, as it is found in the *second* London edition, as well as the *third*, from which the American re-print is taken.

Our limits will not allow us to follow out the Doctor through the other branches of Physicks, contained in this volume. The last section, however, in which he lays down the "Doctrines of fluidity, in relation to animals," presents matter of the highest interest to the medical practitioner, and to the general scholar. He considers these relations apart, under the four following heads. 1st. *Circulation of the Blood;* 2d. *Respiration and Voice;* 3d. *Digestion;* 4th. *Pelvic Phenomena.* He very properly remarks, that no one can understand this section who is ignorant of those which precede it. The mass of truly valuable matter which this section alone contains, will recommend the entire work to the particular attention of the practical enlightened physician. We cannot go into an examination of the ingenious manner in which he explains the mechanism of the circulation. We give a short extract relative to some of the means which the mechanical laws furnish us.

*Diffused pressure,* like that made by rolling a bandage round a whole limb, or by immersing the limb in fluid, must affect the circulation. The veins will be more compressed than the arteries, by reason of the distended force in them being less. Varicose veins, therefore, are usefully supported by a bandage or laced stocking. The reason why this manner of supporting assists so powerfully in the healing of ulcers on the legs may be, that the support affects the capillaries and absorbents as well as the larger vessels.

Poultices, by their weight, produce a soft compression of the parts on which they are applied; and in certain cases, may benefit by mechanically squeezing the excess of blood out of the weakened vessels.

The author has relieved the chronic inflammation of a sprained ancle, by ordering the foot and leg, covered with an oiled silk stocking, to be enclosed in a boot strong enough to support the pressure of quicksilver, and to be then surrounded by this for an hour or more.—The effect is a pressure by the fluid metal on the weak vessels, of one pound to the square inch, for every two inches of the depth of metal above the part. A height of four or five inches gives the relief expected. A much greater elevation would stop the circulation altogether. No bandage can press with uniformity approaching to this action of a fluid.

The effect of continued pressure, in removing morbid tumours of various kinds, is explicable on the same principle. The author doubts not that in such cases, pressure properly managed, would have a much more valuable remedy than is at present generally supposed. The elastic steel half-hoop, with one cushion before and another behind, lately introduced for the relief of hernia, affords an admirable mode of producing a uniform pressure of any desired force upon the female breast.

When a man stands in a bath, with the water up to his chin, there is a pressure of the water upon his body, proportioned every where to the depth (see page 242.) This pressure must produce a considerable effect on the blood vessels of the lower parts of the body. We see in this that a bath must propel the blood from all the external veins of the body towards the cavity of the chest, which the pressure cannot reach: it is this effect which in part causes the feeling of thoracic oppression experienced by persons on first plunging into water, which feeling is usually attributed altogether to the cold.

The old practice of placing a patient in a pit, and surrounding his body with earth or sand, must have had a mechanical action of the kind now contemplated, in addition to any other influence.

### The phenomena of respiration are in many points explicable only by the laws of Physics.

That the air admitted to the chest should have the fullest action on the blood passing there, it was necessary that the spongy mass of lungs in which the blood-vessels ramify, should occupy the whole of the cavity, and be equally distributed. Now while the equable distribution is effected by the uniform elasticity or resilience which belongs to the structure of lung, the complete filling of the cavity is obtained, not by general attachments between the lungs and the ribs or sides of the chest, as might be expected, but by the following means, equally simple, and yet more perfect. The spongy mass of the lungs is completely covered by a strong adherent membrane, called the pleura, as close in its texture as a bladder: between this membrane and a similar lining of the chest, there is no air or empty space, and therefore, in the rising and falling of the ribs during respiration, this membrane remains always in contact with them, just as a bladder put into a bellows as a lining, with its mouth secured around the nozzle, is filled and emptied, and remains in contact with the interior of the bellows, in all its states of dilatation, as if there were attachments in a thousand places. This construction allows the lungs to have a singular freedom of play during all the motions of the body; a freedom farther provided for by their being divided into five portions or lobes, of which three occupy the right side of the chest, and two are in the left, the heart occupying in the left the place of the third. The right and left sides of the chest are formed into cavities quite distinct from each other by the *mediastinum*, a strong membranous partition. The mechanical disposition of the contents of the chest, as now described, is productive of certain consequences which it is important to understand,—for instance,

If a wound be made in one side of the chest so as to admit air, the lungs of that side collapse in obedience to their weight and elasticity; and when the chest afterwards enlarges and diminishes in respiration, air more easily enters and leaves the space around the collapsed lung, through the wound, than it can enter or leave the lung itself through the wind-pipe; because, in the first case, it has no force to overcome, and in the second the elasticity, weight and inertia of the lung oppose. If such a wound, therefore, were made into both sides of the chest at once, although without hurting any part within, the person unless assisted, would die of suffocation, because all the lungs would remain collapsed. The kind of assistance required in such a case, is to press the ribs down so as to empty the chest of air as much as possible, and then to keep the wounds close or covered while the ribs rise again; the air, of course, then enters by the natural road, the only one left, to fill the chest, and it distends the lung, and reaches the blood in the pulmonary vessels as usual. By preventing the breath from escaping by the mouth or nose, while straining with the muscles of the chest, as in the action of blowing, all the air which had entered by any wound in the chest, may be expelled. In Benjamin Bell's system of surgery, which was long the manual of practitioners, from imperfect understanding of this subject, counsel was given the very contrary of that required; and of course, any patient treated accordingly, must have been lost.

In cases of dangerous hæmorrhage from a lung, after a wound in the side, the proper practice is to allow the lung to collapse, as now explained, that the hæmorrhage may be checked; and when the danger is past, the external wound is to be closed, that the natural play of the lung may be re-established. Life may be supported for a time by the lung in one side of the chest.

In cases of hemoptysis, or spontaneous bleeding from the lungs, a disease so often fatal, life might sometimes be saved or prolonged by making an opening between two of the ribs, and allowing the lung to collapse. The affected lung is often clearly pointed out by the circumstance; and the opening, when properly made, would be no more dangerous than in the case where, by a similar opening, water or pus is discharged from the chest.

The same operation has been tried as a forlorn hope in pulmonary consumption. This disease is often limited to the lung of one side, and as the alternate stretching and collapse of the diseased lung during respiration, together with the contact of the air powerfully prevent an ulcer from healing, or inflammation from subsiding. a new chance of recovery is given by allowing the diseased lung to collapse and remain at rest.—Some cases are recorded where cure is said to have followed this operation, and certainly, where the circumstances are favourable for it, and where death must ensue unless it can save, it is worth trying.

When ribs are fractured, it is the practice to put a bandage round the chest, so as for the time to prevent the respiratory motion of the ribs, and the breathing is then performed by the rising and falling of the diaphragm or floor of the chest, as already explained: it bulges into the chest to lessen the cavity, and is again drawn down or flattened, to increase the cavity. Although a person with broken ribs is obliged to submit to this unnatural restraint, it is surely the height of folly to inflict the same on healthy beings, as is yet, however, constantly done among young women, even to the destruction of their health, by the fashion of bracing the body in tight stays.

The force of a healthy chest's action in blowing is equal, as stated in last section, to about one pound on the inch of its surface; that is to say, the chest can condense its contained air with that force, and can therefore blow through a tube, the mouth of which is two feet under the surface of water. In sucking or drawing in air, the power is nearly the same.—In both these actions it is possible to use the cavity of the mouth separately from that of the chest; and the mouth being smaller, with stronger muscles about it in proportion to its size, it can act more strongly. Some men can suck with the mouth so as to make nearly a perfect vacuum, or to lift water nearly thirty feet: In using the blowpipe, an expert operator can keep up an uninterrupted blast by shutting the mouth behind while he inhales, and replenishing it as is required in the intervals.

### We subjoin the following extracts.

In *coughing*, the *glottis* or top of the wind-pipe, by a curious sympathy of parts, is first closed for an instant, during which the chest is compressing and condensing its contained air, and on being then opened, a slight explosion, as it were, of the compressed air takes place, and blows out any irritating matter that may be in the air-passages, just as the burst from the chamber of an air-gun discharges its bullet.—This shutting of the glottis to allow the compression of the air, and its subsequent opening to allow the discharge, may occur at very minute intervals, and many times for one fill of the chest, as is instanced in hooping-cough. The action of cough is often produced by irritation from a cause which cannot be removed by cough, as inflammation of the chest, or tubercles; or even by irritation in a distant part, as when children are teething, or when the stomach is overloaded.

*Sneezing* is a phenomenon resembling cough, only the chest empties itself with great violence at one throe, and chiefly through the nose, instead of through the mouth, as in coughing. The irritation that produces sneezing is generally in the nose ; but as in the case of cough, sneezing may occur from distant sympathies : witness that from worms in the bowels.

*Laughing* consists of quickly repeated expulsions of air from the chest, the voice being heard with them ; but there is never complete closure of the entrance to the wind-pipe as in coughing.

*Crying* differs from laughing almost only in the circumstance of the intervals between the gusts of air being longer. Children laugh and cry in the same breath, and it is often difficult to mark the moment of change.

*Hiccup* is the sudden stopping of a strong inspiration at its commencement.

In *straining* to lift weights, or to make any powerful effort, the air is shut up in the lungs, that there may be steadiness and firmness of the person. At such a time, by the compression and condensation of air around the heart and large blood-vessels, the blood is determined violently outwards from the chest, and often rises to the head, with force that produces giddiness, or even apoplexy ; the eye will become suddenly blood-shot, from a small vessel giving way during straining ; and leech-bites will break out afresh.—the force of this pressure outwards is measured, as already stated, by a column of about two feet of blood ; and this is therefore the measure of the additional arterial and venous tension in the body generally.

*Suffocation* is the name given to what happens when the supply of air to the lungs is in any way prevented. The blood, not then refreshed by the approach of the air, rises to the brain unfit for its purpose, and confusion of thought is immediately produced, soon followed by convulsion and death.

When that happens from mechanical obstruction at the narrow entrance of the wind-pipe, as in croup, by the tenacious films thrown off from the inflamed lining of the air-passages, life may be saved by making a new entrance for air through the wind-pipe lower down in the neck, and keeping it free by a little tube inserted, until the obstruction above be removed.—Where children die with croup, it is frequently not from the violence of the constitutional disease, but from detached films thus accidentally sticking in the narrow entrance of the air passage.

In the cases of strangling and hanging, the tight binding of the rope or ligature crushes inwards the cartilaginous rings of the wind-pipe, and shuts the air-passage. It may also cause apoplexy, by arresting the passage of blood to and from the head ; and there may be dislocation of the cervical vertebræ of the spine.

In *drowning*, communication with the atmosphere is cut off altogether by the supernatant water, and if the chest then expands, it can receive water only instead of air. The nerves and muscles, however, at the entrance of the wind-pipe, being exceedingly irritable, are excited by the contact of any unusual matter, and for a considerable time shut the passage against the intruding liquid. It is partly on this account that after immersion in water and apparent death, when the body is recovered within a moderate time, the life is often preserved.

The second volume contains two parts, of which only one has as yet appeared.

This part is devoted to the subjects of heat and light.

Dr. Arnott first alludes to heat, as being that which causes the difference between summer and winter, and between the gardens of the equator, and the polar wastes.

In the winter of climates, where the temperature is for a time below the freezing point of water, the earth with its waters is bound up in snow and ice, the trees and shrubs are leafless, appearing every where like withered skeletons, countless multitudes of living creatures, owing either to the bitter cold or deficiency of food, are perishing in the snows—nature seems dying or dead; but what a change when spring returns, that is, when heat returns! The earth is again uncovered and soft, the rivers flow, the lakes are again liquid mirrors, the warm showers come to foster vegetation, which soon covers the ground with beauty and plenty. Man, lately inactive, is recalled to many duties; his water-wheels are every where at work, his boats are again on the canals and streams, his busy fleets of industry are along the shores—winged life in new multitudes fills the sky, finny life similarly fills the waters, and every spot of earth teems with vitality and joy. Many persons regard these changes of season as if they came like the successive positions of a turning wheel, of which one necessarily brings the next; not adverting that it is the single circumstance of change of temperature, which does all. But if the colds of winter arrive too early, they unfailingly produce the wintry scene, and if warmth come before its time in spring, it expands the bud and the blossom, which a return of frost will surely destroy. A seed sown in an ice-house never awakens to life.

Again, as regards climates, the earthy matters forming the exterior of our globe, and therefore entering into the composition of soils, are not different for different latitudes,—at the equator, for instance, and near the poles. That the aspect of nature then in the two situations, exhibits a contrast more striking still than between summer and winter, is merely to an inequality of temperature, which is permanent. Were it not for this, in both situations the same vegetables might grow, and the same animals might find their befitting support. But now, in the one, namely, where heat abounds, we see the magnificent scene of tropical fertility: the earth covered with luxuriant vegetation in endless lovely variety, and even the hard rocks festooned with green, perhaps with the vine, rich in its purple clusters. In the midst of this scene, animal existence is equally abundant, and many of the species are of surpassing beauty—the plumage of the birds is as brilliant as the gayest flowers. The warm air is perfume from the spice-beds, the sky and clouds are often dyed in tints as bright as freshest rainbow, and happy human inhabitants call the scene a paradise. Again, where heat is absent, we have the dreary spectacle of polar barrenness, namely, bare rock or mountain, instead of fertile field; water every where hardened to solidity, no rain, nor cloud, nor dew, few motions but drifting snow; vegetable life scarcely existing, and then only in sheltered places turned to the sun—and instead of the palms and other trees of India, whose single leaf is almost broad enough to cover a hut, there are bushes and trees, as the furze and fir, having what may be called hairs or bristles, in the room of leaves. In the winter time, during which the sun is not seen for nearly six months, new horrors are added, *viz.* the darkness and dreadful silence, the cold benumbing all life, and even freezing mercury—a scene into which man may penetrate from happier climes, but where he can only leave his protecting ship and fires for short periods, as he might issue from a diving-bell at the bottom of the ocean. That in these now desolate regions, heat only is wanted to make them like the most favored countries of the earth, is proved by the recent discoveries under ground of the remnant of animals and vegetables formerly inhabiting them, which now can live only near the equator. While winter then, or the temporary absence of heat, may be called the sleep of nature, the more permanent torpor about the poles appears like its death; and when we farther reflect, that heat is the great agent in numberless important processes of chemistry and domestic economy, and is the actuating principle of the mighty steam engine which now performs half the work of society, how truly may heat, the subject of our present chapter, be considered as the life or soul of the universe!

The remarks of our author on the *conducting properties* of various bodies, are possessed of interest, particularly, those which relate to clothing.

Animals required to be protected against the chills of night, and the biting blasts of winter; and some of them which dwell among eternal ice, could not have lived at all, but for a garment which might shut up within it nearly all the heat which their vital functions produced. Now any covering of a metallic, or earthy, or woody nature, would have been far from sufficing; but out of a wondrous chemical union of carbon with the soft ingredients of the atmosphere, those beautiful textures are produced, called fur and feather, so greatly adorning while they completely protect the wearers—textures moreover, which grow from the bodies of the animals, in the exact quantity that suits the climate and season, and which are reproduced when by any accident they are partially destroyed. In warm climates the hairy coat of quadrupeds is comparatively short and thin; as in the elephant, the monkey, the tropical sheep, &c. It is seen to thicken with increasing latitude, furnishing the soft and abundant fleeces of the temperate zones; and towards the poles it is externally shaggy and coarse, as in the arctic bear. In amphibious animals, which have to resist the cold of water as well as of air, the fur grows particularly defensive, as in the otter and beaver. Birds, from having very warm blood, required plenteous clothing, but required also to have a smooth surface, that they might pass easily through the air:—both objects are secured by the beautiful structure of feathers, so beautiful and wonderful, that writers on natural theology have often particularized it as one of the most striking exemplifications of creative wisdom. Feathers, like fur, appear in kind and quantity suited to particular climates and seasons.—The birds of cold regions have covering almost as bulky as their bodies, and if it be warm in those of them which live only in air, in the water-fowl it is warmer still. These last have the interstices of the ordinary plumage filled up by the still more delicate structure called down, particularly on the breast, which in swimming first meets and divides the cold wave. There are animals with warm blood which yet live very constantly immersed in water, as the whale, seal, walrus, &c. Now neither hair nor feathers, however oiled, would have been a fit covering for them; but kind nature has prepared an equal protection in the vast mass of fat or thick oil, which surrounds their bodies—substances which are scarcely less useful to man than the furs and feathers of land animals.

While speaking of clothing, we may remark, that the bark of trees is also a structure very slowly permeable to heat, and securing therefore the temperature necessary to vegetable life.

And while we admire what nature has thus done for animals and vegetables, let us not overlook her scarcely less remarkable provision of ice and snow, as winter clothing for the lakes and rivers, for our fields and gardens. Ice, as a protection to water and its inhabitants, was considered in vol. i. in the explanation of why, although solid, it swims on water. We have now to remark that snow, which becomes as a pure white fleece to the earth, is a structure which resists the passage of heat nearly as much as feathers. It, of course, can defend only from colds below 32 degrees or the freezing point; but it does so most effectually, preserving the roots and seeds, and tender plants during the severity of winter. When the green blade of wheat and the beautiful snow-drop flower appear in spring rising through the melting snow, they have recently owed an important shelter to their wintry mantle. Under deep snow, while the thermometer in the air may be far below zero, the temperature of the gronnd rarely remains below the freezing point. Now this temperature, to persons some time accustomed to it, is mild and even agreeable. It is much higher than what often prevails for long periods in the atmosphere of the centre and north of Europe. The Laplander, who during his long winter lives under ground, is glad to have additionally over head a thick covering of snow. Among the hills of the west and north of Britain, during the storms of winter, a house or covering of snow frequently preserves the lives of travellers, and even of whole flocks of sheep, when the keen north wind catching them unprotected, would soon stretch them lifeless along the earth.

Again,

It is the difference of conducting power in bodies which is the cause of a very common error made by persons in estimating the temperature of bodies by the touch. In a room without a fire all the articles of furniture soon acquire the same temperature; but if in winter, a person with bare feet were to step from the carpet to the wooden floor, from this to the hearth stone, and from the stone to the steel fender, his sensation would deem each of these in succession colder than the preceding. Now the truth being that all had the same temperature, only a temperature inferior to that of the living body, the best conductor, when in contact with the body, would carry off heat the fastest, and would therefore be deemed the coldest. Were a similar experiment made in a hot house, or in India, while the temperature of every thing around were 98 deg., *viz.* that of the living body, then not the slightest difference would be felt in any of the substances: or lastly, were the experiment made in a room where by any means the general temperature were raised considerably above blood-heat, then the carpet would be deemed considerably the coolest instead of the warmest, and the other things would appear hotter in the same order in which they appeared colder in the winter room. Were a bunch of wool and a piece of iron exposed to the severest cold of Siberia, or of an artificial frigorific mixture, a man might touch the first with impunity (it would merely be felt as rather cold;) but if he grasped the second, his hand would be frost-bitten and possibly destroyed: were the two substances on the contrary, transferred to an oven, and heated as far as the wool would bear, he might again touch the wool with impunity: it would then be felt as a little hot, but the iron would burn his flesh. The author has entered a room where there was no fire, but where the temperature from hot air admitted was sufficiently high to boil the fish, &c., of which he afterwards partook at dinner; and he breathed the air with very little uneasiness. He could bear to touch woollen cloth in this room, but no body more solid.

The foregoing considerations make manifest the error of supposing that there is a positive warmth in the materials of clothing. The thick cloak which guards a Spaniard against the cold of winter is also in summer used by him as protection against the direct rays of the sun: and while in England flannel is our warmest article of dress, yet we cannot more effectually preserve ice than by wrapping the vessel containing it in many folds of softest flannel.

In every case where a substance of different temperature from the living body touches it, a thin surface of the substance immediately shares the heat of the bodily part touched—the hand generally; and while in a good conductor, the heat so received quickly passes inwards or away from the surface, leaving this in a state to absorb more, in the tardy conductor the heat first received tarries at the surface, which consequently soon acquires nearly the same temperature as the hand, and therefore, however cold the interior of the substance may be, it does not cause the sensation of cold. The hand on a good conductor has to warm it deeply, a slow conductor it warms only superficially. The following cases farther illustrate the same principle. If the ends of an iron poker, and of a piece of wood of the same size, be wrapped in paper, and then thrust into a fire, the paper on the wood will begin to burn immediately, while that on the metal will long resist:—or if pieces of paper be laid on a wooden plank and on a plate of steel, and then a burning coal be placed on each, the paper on the wood will begin to burn long before that on the plate. The explanation is, that the paper in contact with the good conductor loses to this so rapidly the heat received from the coal, that it remains at too low a temperature to inflame, and will even cool to blackness the touching part of the coal; while on the tardy conductor the paper becomes almost immediately as hot as the coal. It is because water exposed to the air cannot be heated beyond 212 deg. that it may be made to boil in an egg-shell or a vessel made of paper, held over a lamp, without the containing substance being destroyed; but as soon as it is dried up, the paper will burn and the shell will be calcined, as the solder of a common tinned kettle melts under the same circumstances. The reason why the hand judges a cold liquid to be so much colder than a solid of the same temperature is, that from the mobility of the liquid particles among themselves, those in contact with the hand are constantly changing. The impression produced on the hand by very cold mercury is almost insufferable, because mercury is both a ready conductor and a liquid. Again, if a finger held motionless in water feel cold, it will feel colder still when moved about; and a man in the air of a calm frosty morning does not experience a sensation nearly so sharp as if with the same temperature there be wind. A finger held up in the wind discovers the direction in which the wind blows by the greater cold felt on one side, the effect being still more remarkable, if the finger is wetted.—If a person in a room with a thermometer, were with a fan or bellows to blow the air against it, he would not thereby lower it, because it had already the same temperature as the air, yet the air blown against his own body would appear colder than when at rest, because, being colder than his body, the motion would supply heat-absorbing particles more quickly. In like manner, if a fan or bellows were used against a thermometer hanging in a furnace or hot house, the thermometer would suffer no change, but the air moved by them against a person would be distressingly hot, like the blasting sirocco of the sandy deserts of Africa. If two similar pieces of ice be placed in a room somewhat warmer than ice, one of them may be made to melt much sooner than the other, by blowing on it with a bellows. The reason may here be readily comprehended why a person suffering what is called a cold in the head, or catarrh from the eyes and nose, experiences so much more relief on applying to the face a handkerchief of linen or cambric than one of cotton:—it is, that the former by *conducting* readily absorbs the heat and diminishes the inflammation, while the latter, by refusing to give passage to the heat, increases the temperature and the distress. Popular prejudice has held that there was a poison in cotton.

Perhaps we might remark in two or three places, a tendency to repetition in the author, evidently shewing marks of haste and inattention. At page 34, he tells us "a black stone," &c., and then in page 38, "as a black earthen," &c.

The effect of caloric in regulating the climate in elevated regions, is prettily expressed.

We have just said, that even at the equator, where the average temperature near the sea is 84 deg., water will be frozen when carried to an elevation of 15,000 feet. A line, therefore, traced on a mountain at this level, would divide the portion of it destined to sleep under lasting ice and snow from the portion below covered with green herbage. This line, wherever found, is called the *snow line*, or *line of perpetual congelation*. At the equator it is high in the atmosphere, because there is a difference of about 50 deg. between the average temperature of the country and the freezing point of water, viz. the difference between 84 deg. and 32 deg., and an elevation of 15,000 feet corresponds to this difference; but in a progress towards the poles, the line is met with gradually nearer the earth, as the difference in question is less. In Switzerland it is at 6,500 feet above the sea; in Norway, it is below 5,000. With respect to the line of congelation, it is farther to be remarked, that in tropical countries, because the temperature of the air is nearly uniform during the whole year, the line or limit of frost and snow is distinct and unvarying, that is to say, is narrow, particularly where the acclivity is considerable; but, in countries to the north and south, which experience strong contrast of summer and winter, the line becomes broad and less evident; because, in the hot season much snow is melted, or half melted, above what may be called an average line, while in winter much snow and ice are accumulated below this, to be melted again when summer returns.

In the breadth of the line of congelation for changeable climates, we have the reason of the formation of what are called glaciers, around snow-capped mountains situated in such climates, and around such only. The snow near the upper part of the broad line having been only softened or half-thawed in the preceding summer, becomes in winter, almost as solid as ice, and in the succeeding summer vast masses of this, detached by the action of the sun, and of the central heat of the earth, spreading outwards, and loaded with more recent accumulation of snow, are constantly falling down into the valleys beneath, where, being accumulated, and the crevices filled up with snow or with water, which hardens to ice, they form at last the huge glaciers, or seas of ice—*mers de glace*, which render certain regions so remarkable.—The falling of such masses (called in Switzerland *avalanches*,) is what renders the ascent to snow-clad mountains terrific and dangerous.—Around Mont Blanc, in the awful solitudes of the elevated valleys, the avalanches are thundering down almost without interruption during the whole summer—in which season only the attempt to ascend the mountain can be made, and a pistol-shot, or any considerable agitation of the air, may suffice to set loose masses that will sweep away a whole convoy. Beneath glaciers there is always going on a melting of that part of the ice which is in contact with the earth, and hence a stream of water constantly issues from the bed of every glacier. These streams, in Switzerland, are the beginnings of the magnificent rivers, the Rhine and the Rhone. Like the avalanches breaking loose in summer among the mountains, there are in the polar seas vast masses of ice detached from the shores, and which afterwards move into warmer seas to be melted. These often become, to the arctic bear, rafts, on which, to his surprise, he finds himself voyaging into new latitudes, to be left at last adrift in the wide ocean when his ship has vanished from beneath him.

Although the proofs are not so immediately apparent, the line of congelation exists as truly every where in the open sky, over sea and plains, as where there are mountain heights to wear its livery; and considerably below the line, the cold, aided by electrical agency, is sufficient to produce, in the form of mist or clouds, a deposition from the air of the watery vapour contained in it. There is thus in nature an admirable provision to shade the earth at proper times from the too powerful rays of the sun, or to supply rain as wanted, without the transparency of the inferior regions of the atmosphere being much affected. As the watery vapour, rising from sea or lake, and invisibly diffused in the atmosphere, can only reach to the height where the cold is great enough to condense it, the clouds may in general be regarded as the top of that atmosphere of watery vapour, or aeriform water, which is always mixed more or less with the atmosphere of mere air; and as the quantity of watery vapour which can exist invisibly in a given space, depends altogether on the intensity of heat present, the clouds in a humid atmosphere will be low, and in a dry atmosphere will be high, or there may be none. An aeronaut mounting in his balloon through a clear sky, often approaches a dense cloudy stratum to plunge into it, and for a time be surrounded with gloom almost of night; the face of earth being hidden from him below, while the heavenly bodies are equally veiled from him above; but rising still higher, he again emerges to brightness, and looks down upon the fleecy ocean rolled in mountain heaps beneath him, as the climber to a lofty peak may look down from the ever pure atmosphere around it on the inferior region of clouds and storms.

The diminished temperature of air in the higher regions of the atmosphere, often enables the natives of temperate climates, when led by circumstances to reside in tropical countries, where their health may suffer from the heat, to find near at hand, on some mountain height, the congenial temperature of their early homes. The author once, during a visit to the recently inhabited island of Penang, in the strait of Malacca, examined this fact with pleasure not readily forgotten. The centre of the island is occupied by a lofty mountain ridge thickly wooded, on the northern summit of which a few residences, visible from the sea-shore like eagles' nests on a cliff, had just been constructed. Towards these, one morning at sun-rise, on an active little horse of the country, and along a tolerable road, he began to climb from the hot plain below. At first there were around him purely tropical objects, inspiring tropical feelings,—the latter, modified indeed, by the reflection that his track lay through a forest, into which until lately the foot of man had never ventured, and where the trees, nursed through ages to their greatest growth, and the stupendous precipices, and the sublime water-fall, &c., had so recently been exposed to human observation; but, as he gradually ascended, the character of the vegetation was perceived to be changing, and the air was becoming so light and cool as irresistibly to awaken in him thoughts of distant England—nay, almost the illusion of his being there. At last, however, the summit being reached, where a clear space opened to view the whole country around, the attention was quickly recalled to the fervid land of the sun. The elevation is so great that at first the eye takes account of only the grander features of the scene, and such nearly as might be met with on a Grecian or Italian shore:—the expanse of sunny water in that beautiful strait, stretching so far north and south, the opposite continental shore with its river winding seaward across the plain, the town and the roadstead near it crowded with ships, which appeared only as specks in a wide-spread map, &c.; but on closer inspection, and particularly with the aid of the telescope, were descried the rich groves of cocoa nut and banana, the plantations of spice, and cotton, and sugar cane, the tawny laborers, the bamboo dwellings, the fanciful canoes or prows, in a word, every object bespeaking the torrid zone. Such then is the scene, which even under the equator, an invalid by climbing a hill may place under his eye, and where the thermometer near him stands as in an English month of May.

We pass over the capacity of bodies for caloric, and the general principles of latent heat, and the effects of caloric on chemical changes, whether producing them, or produced by them, to its influence on inanimate nature, whether vegetable or animal.

The influence which heat exerts on inanimate nature, is more immediately and completely perceived by the common mind, than its influence on beings which have life. Thus to all it is obvious, that the contrast between a winter and summer landscape, is owing chiefly to the effect of heat on the water of the landscape;—that during its absence in winter, there is the dry barren deformity of accumulated ice and snow, covering every thing, the roads impassable, the rivers bound up, perhaps hidden, the air deprived of moisture, and loaded often with powdery drift; and that when warmth comes, the living streams again appear, gliding their way, the cascades pour, the rills murmur, the canals once more offer their bosoms to the boats of commerce, the lake and pool again show their level face, reflecting the glories of the heavens, and the genial shower falls upon the bosom of the softened earth, become ready to receive the spade or the ploughshare. Now this change is not at all greater than what happens to a winter tree acted upon by the warmth of spring. To take another instance from inanimate nature, it may be said with truth, that heat applied to the cold boiler of a steam-engine, is the cause of all its succeeding motions; of the heaving of its beam and pumps, the opening and shutting of its valves, the turning of its wheels and its ultimate performance of work, as of spinning, or weaving, or grinding, or propelling vehicles by land and water; but as truly may it be said, that heat coming to a seed, which has lain cold for ages, is the cause of its immediate germination and growth, or coming to a lately frozen tree, is the cause of the rising of its sap, the new budding and unfolding of its leaves and blossoms, the ripening of its fruit. And what is true of one seed or tree, is true of the whole of the vegetable creation. When the warm gales of spring have once breathed on the earth, it soon becomes covered, in field and in forest, with its thick garb of green, and soon opening flowers or blossoms every where breathe back again a fragrance to heaven.—Among these the heliotrope is seen always turning its beautiful disk to the sun, and many delicate flowers only open their leaves to catch the direct solar ray, but close them often even when a cloud intervenes, and certainly when the chills of night approach. On the sunny side of a hill, or in the sheltered crevice of a rock, or on a garden wall with warm exposure, there may be produced grapes, peaches, and other delicious fruits, which will not grow in situations of an opposite character—all acknowledging heat as the immediate cause, or indispensable condition of vegetable life.

But among animals too, the effects of heat are equally remarkable. The dread silence of winter, for instance, is succeeded in spring by one general cry of joy. Aloft in the air the lark is every where carolling; and in the woods and shrubberies, a thousand little throats are similarly pouring forth their songs of gladness—during the day, the thrush and blackbird near our dwellings, are heard above the rest, and with the evening comes the sweet nightingale—for all of which it is the season of love and of exquisite enjoyment. And it is equally so for animal nature generally: in favored England, for instance, in April and May the whole face of the country resounds with lowings and bleatings and barkings of joy. And even man, the master of the whole, and whose mind embraces all times and place, is far from being insensible to this change of season. His far-seeing reason, of course, draws delight from the anticipation of autumn, with its fruits; and his benevolence rejoices in the happiness observed among all inferior creatures; but independently of these considerations, on his own frame the returning warmth exerts a direct influence. In early life, when the natural sensibilities are yet fresh and unaltered by the habits of artificial society, spring to man is always a season of delight. The eyes brighten, the whole countenance is animated, and the heart feels as if new life were come, and has longings for fresh objects of endearment.—Of those who have passed their early years in the country, or among the charms of nature, as contrasted with the arts of cities, there are few who, in their morning walks in spring, have not experienced without very definite cause, a kind of tumultuous joy, of which the natural expression would have been, how good the God of nature is to us! Spring is a time when sleeping sensibility is roused to feel that there lies in nature more than the grosser sense perceives. The heart is then thrilled with sudden extacy, and wakes to aspiration of sweet acknowledgment.

### The functions of animal life are a source of heat.

It is one of the remarkable facts in nature, that living animal bodies, and to a certain degree, living vegetables also, have the property of maintaining in themselves a peculiar temperature, whether surrounded by bodies that are hotter or colder than they. Captain Parry's sailors, during the polar winter, where they were breathing air that could freeze mercury, still had the natural warmth in them of 98 deg. Fahrenheit; and the inhabitants of India, where the same thermometer stands sometimes at 115 deg. in the shade, have their blood at no higher a temperature.

It was at one time the favorite explanation of this, that animal heat was produced in the lungs, during respiration, from the oxygen then admitted. This oxygen combines with carbon from the blood, and becomes carbonic acid as in combustion, and it was supposed to give out a portion of its latent heat, as in actual combustion; which heat being then spread over the body by the circulating blood, maintained the temperature.—We now know, that if such a process assist, which it probably does,—for the animal heat has generally a relation to the quantity of oxygen expended in any particular case, and when an animal being already much heated needs no increase, very little oxygen disappears,—still much of the effect is dependent on the influence of the nerves, either directly or indirectly, through the vital functions governed by them. Mr. Brodie, in his important experiments upon the subject, found that although in animals apparently dead from injury done to the nervous system, he could artificially continue the action of respiration, with the usual formation of carbonic acid, still the temperature fell very quickly. The maintenance of low temperature in an animal immersed in air hotter than itself, is partly attributable to the copious perspiration and evaporation which then take place, and which absorb into the latent form the excess of heat then existing. Perspiration, both from the skin and internal surface of the lungs, occurs generally in proportion to the excess of heat. Dogs and other animals, when much heated, as they cannot throw off or diminish their natural covering, increase the evaporating surface by protruding a long humid tongue.

The power in animals of preserving their peculiar temperature has its limits. Intense cold coming suddenly upon a man who has not sufficient protection, first causes a sensation of pain, and then brings on an almost irresistible sleepiness, which if indulged would be fatal. Sir Joseph Banks having gone on shore one day near the cold Cape Horn, and being fatigued, was so overcome by the feeling mentioned, that he entreated his companions to let him sleep for a little while. His prayer granted, might have allowed that sleep to come upon him which ends not—the sleep of death! as, under similar circumstances, it came upon so many thousands of the army which Bonaparte led into Russia, and lost there during the disastrous retreat through the snows. Bonaparte's celebrated bulletin allowed, that in one night, when the thermometer of Reaumur stood at 19 deg. below zero, 30,000 horses died! Cold in inferior degrees, and longer continued, acting on persons imperfectly protected by clothing, &c., induces a variety of diseases, which destroy more slowly. A great excess of heat, again, may at once excite a fatal apoplexy, and heat in inferior degrees, but long continued, may cause those fevers, &c., which prevail in warm climates, and which are so destructive to strangers in these climates.

Each species of animal has a peculiar temperature natural to it, and in the diversity are found creatures fitted to live in all parts of the earth, what is wanting in internal bodily constitution, being found in the admirable protecting covering which nature has provided for them—covering which grows from their bodies, with form of fur or feather, in the exact degree required, and even so as in the same animal to vary with climate and season. Such covering, however, has been denied to man; but the denial is not one of unkindness:—it is the indication of his superior nature and destinies. Godlike reason was bestowed on man, by which he subjects all nature to his use, and he was left to clothe himself.

The human race is naturally the inhabitant of a warm climate, and the paradise described as Adam's first abode, may be said still to exist over vast regions about the equator. There the sun's influence is strong and uniform, producing a rich and warm garden, in which human beings however ignorant of the world which they had come to inhabit, would have their necessities supplied almost by wishing. The ripe fruit is there always hanging from the branches; of clothing there is required only what moral feelings may dictate, or what may be supposed to add grace to the form; and as shelter from the weather, a few broad leaves spread on connected reeds will complete an Indian hut. The human family, in multiplying and spreading in all directions from such a centre, would find to the east and west, only the lengthened paradise, with slightly varying features of beauty; but to the north and south, the changes of season, which make the bee of high latitudes lay up its winter store of honey, and send migrating birds from country to country in search of warmth and food, would also rouse man's energies to protect himself. His faculties of foresight and contrivance would come into play, awakening industry; and, as their fruits, he would soon possess the knowledge and the arts which secure a happy existence in all climates, from the equator almost to the pole. It is chiefly because man has learned to produce at will, and to control the wonder-working principle of heat, that in the rude winter, which seems the death of nature, he, and other tropical animals and plants which he protects, do not in reality perish—even as a canary bird escaped from its cage, or an infant exposed among the snow-hills. By producing heat from his fire, he obtains a novel and most pleasurable sort of existence; and in the night, while the dark and freezing winds are howling over his roof, he basks in the presence of his mimic sun, surrounded by his friends and all the delights of society, while in his store-rooms, or in those of merchants, at his command, he has the treasured delicacies of every season and clime. He soon becomes aware, too, that the dreary winter, instead of being a curse, is really in many respects a blessing, by arousing him from the apathy to which the eternal serenity of a tropical sky so much disposes. In climates where labour and ingenuity must precede enjoyment, every faculty of mind and body is invigorated; and hence the sterner climates form the perfect man. It is in them that the arts and sciences have reached their present advancement, and that the brightest examples have appeared of intellectual and moral excellence.

